# Precision Proteomic Profiling of Systemic Lupus Erythematosus—Correlating Disease Activity and Complement Levels with Clinical Phenotypes

**DOI:** 10.3390/biomedicines14061408

**Published:** 2026-06-22

**Authors:** Jacob Skallerup, Christopher Aboo, Dorte B. Bekker-Jensen, Katherine Tran, Jie Ren, Malene Møller Jørgensen, Jonathan M. Blackburn, Anne Troldborg, Allan Stensballe

**Affiliations:** 1 Department of Health Science and Technology, Aalborg University, 9260 Gistrup, Denmark; jacobsa@hst.aau.dk (J.S.);; 2 Sino-Danish Center for Education and Research, University of Chinese Academy of Sciences, Beijing 100190, China; 3 Evosep Biosystems, 5230 Odense, Denmark; dbj@evosep.com; 4 SCIEX, Concord, Vaughan, ON L4K 4V8, Canada; katherine.tran@sciex.com; 5 China National Center for Bioinformation, Beijing 100101, China; renjie@big.ac.cn; 6 CAS Key Laboratory of Genomic and Precision Medicine, Collaborative Innovation Center of Genetics and Development, School of Future Technology, Beijing Institute of Genomics, Chinese Academy of Sciences, Beijing 100101, China; 7 University of Chinese Academy of Sciences, Beijing 100049, China; 8 Institute for Stem Cell and Regeneration, Chinese Academy of Sciences, Beijing 100101, China; 9 Department of Clinical Immunology, Aalborg University Hospital, 9000 Aalborg, Denmark; maljoe@rn.dk; 10 Department of Clinical Medicine, Aalborg University, 9220 Aalborg, Denmark; 11 Institute of Infectious Disease & Molecular Medicine, Faculty of Health Sciences, University of Cape Town, Cape Town 7925, South Africa; jonathan.blackburn@uct.ac.za; 12 Department of Rheumatology, Aarhus University Hospital, 8200 Aarhus, Denmark; annetrol@rm.dk; 13 Department of Biomedicine, Aarhus University, 8000 Aarhus, Denmark; 14 Department of Clinical Medicine, Aarhus University, 8200 Aarhus, Denmark; 15 Australian National Phenome Center, Australian National Phenome, Murdoch University, 5 Robin Warren Drive, Perth, WA 6150, Australia

**Keywords:** SLE, autoantibody, protein array, proteomics, KREX

## Abstract

**Background/Objectives:** Systemic lupus erythematosus (SLE) is characterized by diverse clinical presentations and complex immunological mechanisms. This study aimed to characterize patient serology associated with disease activity scored using the systemic lupus erythematosus disease activity index (SLEDAI) and investigate the molecular signature of complement activation (measured through C3dg, a complement breakdown product) in SLE patients utilizing high-throughput mass spectrometry and autoantibody profiling. **Methods:** Plasma samples from 39 SLE patients in four mutually exclusive groups based on either disease activity scores (high/low SLEDAI) or complement activation levels (high/low C3dg) were analyzed using rapid LC-MS/MS, followed by unsupervised and supervised protein expression analysis. Complement activation was evaluated by measuring C3dg levels, and disease activity was scored using SLEDAI. Autoantibody reactivities were profiled using global autoantibody protein microarrays. Data are available via ProteomeXchange with identifier PXD066214. **Results:** Differential proteomic analyses revealed 25 proteins associated with SLE disease activity (high vs. low SLEDAI scores) and 25 proteins linked to complement activation levels (high vs. low C3dg). Enriched pathways indicated that adaptive immune response, classical complement activation, and immunoglobulin production correlated with disease activity, while complement activation and coagulation cascades were primarily associated with complement activation levels. Autoantibody profiling highlighted distinct reactivity patterns between subgroups, suggesting varying degrees of immune-mediated tissue damage. **Conclusions:** In this study, disease activity and complement activation markers were associated with overlapping yet non-identical plasma proteomic patterns in SLE. These findings support the feasibility of rapid mass spectrometry-based proteomics and autoantibody profiling for generating candidate molecular signatures in SLE. These findings serve as exploratory signatures that require validation in larger independent cohorts before they can be considered for clinical stratification and decision-making.

## 1. Introduction

Systemic lupus erythematosus (SLE) is a chronic autoimmune disorder characterized by the presence of hallmark autoantibodies targeting nuclear components through anti-nuclear antibodies (ANA). The clinical manifestations are widely heterogeneous, hence SLE is characterized as a multisystem disease, with the skin, kidneys, and joints being among the most commonly affected organs [1]. While ANA positivity is relatively common in the general population, it does not always result in the development of SLE [1]. Thus, additional factors play a role in the transition from autoimmunity to manifestation of clinical symptoms. The exact mechanisms of this transition remain unclear; however, development of the disease is believed to be the result of a combination of genetic predisposition and environmental triggers [1,2,3]. This is confirmed in studies that have shown a ~25% co-occurrence of SLE in monozygotic twins, indicating a strong but not exclusive genetic component to disease development [1].

SLE is diagnosed based on a combination of clinical symptoms and laboratory assays; however, no generally accepted diagnostic criteria or tests for SLE diagnosis exist [1]. The diagnosis is clinical, but for research purposes patients are classified based on classification criteria developed by the EULAR/American College of Rheumatology (ACR). The classification criteria were developed primarily for clinical trial classification of patients, and thus are focused on obtaining homogenous groups of patients while ensuring high specificity for SLE at the cost of lower sensitivity (ACR SLE criteria have 86% sensitivity and 93% specificity). When classification criteria are mistakenly applied for diagnostic purposes, some patients may fall short of the criteria and may wrongly be diagnosed as not having SLE, especially at early stages of disease, potentially worsening patient prognosis due to lack of early intervention. The current SLE classification criteria in routine use, SLICC 2012 and ACR/EULAR 2019, both include complement measurements as part of the laboratory/immunologic assessment. In contrast, the older ACR criteria did not specify individual complement factor measurements, despite the well-established role of the complement system in SLE pathogenesis [1,4].

The systemic lupus erythematosus disease activity index (SLEDAI) is a clinical scoring system used to assess and quantify disease activity in patients with SLE [5]. It includes a range of clinical and laboratory criteria, such as symptoms affecting the skin, joints, kidneys, central nervous system, and laboratory findings like complement levels and presence of autoantibodies, e.g., dsDNA. Scores have a theoretical range from 0 to 105. Disease activity can be categorized as no activity (0), mild activity (1–5), moderate activity (6–10), high activity (11–19), and very high activity (≥20) [6]. The index guides clinicians in monitoring disease progression and treatment effectiveness, and can aid clinical decision-making. Quantification of specific complement components C3 and C4 has long been common practice in clinics to aid diagnosis [4]. In 2012, Systemic Lupus International Collaborating Clinics (SLICCs) released updated classification criteria which included C3, C4 and CH50, a measurement of total complement activity, among the immunological criteria for SLE classification [7]. However, hypocomplementemia alone is not a reliable marker for distinguishing SLE patients from healthy controls, and studies have found that C3 levels alone do not consistently distinguish SLE patients from healthy individuals and, furthermore, are not consistently correlated with disease activity [4,8]. Consequently, rather than relying on complement levels as a diagnostic criterion, quantifying split products of complement activation such as C3dg has been proposed as a more direct measurement of actual complement activation [4,8,9]. The diagnostic value of C3dg has previously been demonstrated by Troldborg et al., who showed that C3dg levels differentiated SLE patients from controls with an AUC of 0.96 compared to an AUC of 0.52 for conventional C3 levels [4]. However, while the discriminatory value of C3dg has been established, less is known about the plasma proteomic phenotype and biological processes reflected by differing C3dg levels. Autoantibodies associated with SLE are often present in patients with a variety of autoimmune diseases, affecting the diagnosis and differentiation of the diseases [10,11]. A variety of biofluid autoantibodies, such as anti-dsDNA, anti-nucleosome, anti-Sm, anti-ribosomal P and anti-C1q antibodies, have been shown to be prevalent in patients with SLE. The detection of autoantibodies is crucial for assisting in the diagnosis and assessment of SLE activity [12]. The presence and levels of these autoantibodies are highly correlated with disease progression [13].

In this study, we compared the molecular profiles of SLE patients stratified by SLEDAI and C3dg levels using high-throughput LC-MS/MS-based proteomics capable of analyzing 500 samples per day (SPD) in combination with global autoantibody profiling against 1631 antigens using protein microarrays. We investigated candidate molecular and biological signatures associated with SLEDAI-based disease activity and C3dg-defined complement activity.

## 2. Materials and Methods

### 2.1. Cohort and Criteria for Participation

This cross-sectional cohort study was conducted in accordance with the Declaration of Helsinki. The study was approved by The Danish Data Protection Agency and The Central Denmark Region Committees on Health Research Ethics (case number: 1-10-72-214-13; date of final approval: 1 November 2013). Inclusion criteria were age over 18 years, written informed consent, and fulfillment of at least four out of 11 ACR classification criteria for SLE. Patients were excluded if they were receiving treatment for active infection or cancer.

This study includes 39 SLE patients selected from a larger cohort of 169 patients (Troldborg et al. 2018) [4] based on SLEDAI scores and C3dg levels. Patients were stratified into four groups representing each parameter: (1) high disease activity (SLEDAI > 10; *n* = 10), (2) low disease activity (SLEDAI < 5; *n* = 11), (3) high complement activation (elevated C3dg; *n* = 9), and (4) low complement activation (low C3dg; *n* = 9). Importantly, group assignment was performed independently for each parameter; SLEDAI-based groups were defined regardless of C3dg levels, and C3dg-based groups were defined regardless of SLEDAI scores. Each patient was included in only one subgroup, resulting in four mutually exclusive groups. This design enabled assessment of proteomic signatures associated with disease activity and complement activation. Patients were selected from the available parent cohort to represent the extremes of each parameter (high vs. low SLEDAI and high vs. low C3dg).

Blood samples from these patients were collected by laboratory technicians at Aarhus University Hospital during routine blood testing. The samples were processed within one hour from collection. The plasma used in this study was obtained by centrifugation in EDTA tubes (Alere Inc., Waltham, MA, USA, cat. no. 367525) for 10 min at 2000× *g* after which the plasma was immediately aliquoted into tubes and frozen at −80 °C.

### 2.2. Sample Preparation for Mass Spectrometry

The protein content from the plasma samples was isolated and digested to peptides using a filter-aided sample preparation (FASP) approach. In brief, plasma protein concentrations were determined by measuring absorbance at 280 nm (A280) using a NanoDrop ND-1000 spectrophotometer (Thermo Fisher Scientific, Waltham, MA, USA). A plasma volume containing 100 µg of protein was reduced and alkylated in 10 mM Tris(2-carboxyethyl)phosphine hydrochloride (TCEP; Sigma-Aldrich, St. Louis, MO, USA, cat. no. 646547) and 25 mM 2-chloroacetamide (CAA; Sigma-Aldrich, St. Louis, MO, USA, cat. no. C0267) for 30 min at room temperature. Following reduction and alkylation, the samples were processed through several filtration and centrifugation steps as described in detail by Bennike et al. [14]. The proteins were then digested using trypsin (Promega, Madison, WI, USA) at a 1:100 enzyme-to-protein ratio (*w*/*w*) overnight at 37 °C. Following digestion, the resulting peptides were collected by centrifugation through the filter and extracted by solvent phase transfer, as described by Masuda et al. [14,15]. Finally, the samples were dried by vacuum centrifugation and reconstituted in a loading buffer for mass spectrometry analysis (0.1% formic acid, 0.1% trifluoroacetic acid, and 2% acetonitrile).

### 2.3. Mass Spectrometric Analysis

Approximately 200 ng protein from each sample was loaded in quadruplicate onto Evotip Pure tips (Evosep Biosystems, Odense, Denmark), which function as desalting trap columns, according to the manufacturer’s instructions. The loaded samples were then placed on the autosampler of an Evosep One liquid chromatography (LC) system (Evosep Biosystems, Odense, Denmark). Samples were eluted onto a 4 cm Evosep Endurance C18 column (Evosep Biosystems, Odense, Denmark). The LC system was coupled to a ZenoTOF 7600 (SCIEX, Singapore, Singapore) mass spectrometer operated in positive ion mode using data-independent acquisition (Zeno SWATH) with 56 variable isolation windows spanning an *m*/*z* range of 400 to 750. Fragment ions were generated by collision-induced dissociation (CID) and analyzed in an *m*/*z* range of 140 to 1750. The effective gradient length was 2.2 min with a total cycle time of 2.9 min, enabling analysis of 500 samples per day (500 SPD) using a standardized Evosep LC method.

### 2.4. Data Processing and Analysis

Following data acquisition, the raw MS files were processed and analyzed using Spectronaut (version 18.7.240325.55695; Biognosys AG, Schlieren, Switzerland). The raw spectral data were matched against a reviewed human protein library (UniProt UP000005640_9606 from UniProt, downloaded 8 January 2024). The search was performed using the DirectDIA+ Deep workflow with default settings including 1% false-discovery-rate thresholds for proteins, peptides, and peptide-spectrum matches (PSMs), a maximum of two missed cleavages, variable modifications in the form of acetylation of protein N-terminals and oxidation, and fixed carbamidomethylation of cysteines.

After processing in Spectronaut, the protein quantification report was downloaded and processed in Perseus (version 1.6.10.0; Max Planck Institute of Biochemistry, Martinsried, Germany) [16]. Protein identifications were excluded if the protein was not observed in 50% of all acquisitions and if they were identified by fewer than two unique peptides. The protein intensities across the quadruplicate acquisitions were averaged, and differential expression analyses were performed to compare protein expression across groups; high versus low activity and high versus low C3dg. The differential expression analysis was performed using two-sided *t*-tests. Due to the relatively small sample size and the exploratory nature of the study, proteins with raw *p*-values < 0.05 were considered statistically significant, and no multiple-testing correction was applied. Accordingly, these findings should be regarded as hypothesis-generating and require validation in independent cohorts.

The filtered and processed data was exported, plotted using Python (version 3.11; Python Software Foundation, Wilmington, DE, USA), and further analyzed in R (version 4.4.0; R Foundation for Statistical Computing, Vienna, Austria) using the mixOmics package (version 6.27.0) [17]. Two sparse Partial Least Squares—Discriminant Analysis (sPLS-DA) models were created with the aim of selecting the most discriminative proteins between SLE groups in this study; one of the models compares high- and low-SLEDAI patients, and the other compares the high- and low-C3dg groups. In short, the models were tuned to determine the optimal number of components and features of each component to base the model on. To balance model performance, interpretability, and the risk of overfitting due to our relatively small number of samples, we limited the number of features the model was allowed to use for each component to a range between 1 and 30. Model performance was assessed using repeated M-fold cross-validation (10-fold (activity), 9-fold (C3dg), 75 repeats) implemented in the perf() function from the mixOmics package, reporting a balanced error rate (BER). Given the small subgroup size, the sPLS-DA models were used as exploratory feature-selection tools, and selected features should be interpreted cautiously and require validation in independent cohorts.

### 2.5. Autoantibody Profiling of Patient Groups

A subset of 20 of the 39 plasma samples included in the LC-MS/MS analysis were combined in four separate pools, with each pool representing one of the four patient subgroups (*n* = 5 per group, randomly selected from each SLE group). Each of the four pools were analyzed for autoantibody reactivities using immunome protein microarrays (SomaLogic, Boulder, CO, USA) that contain 1631 recombinant, natively folded human proteins expressed through KREX technology spotted on the arrays in quadruplicate.

The sample analysis was performed in accordance with the manufacturer’s instructions. In short, the microarrays were washed in serum albumin buffer (SAB) containing 0.1% Triton X-100 and 0.1% bovine serum albumin in phosphate-buffered saline, after which they were incubated for two hours with the plasma pools diluted 1:200 in SAB. Sample incubation was followed by a series of washing steps in SAB before the addition of polyclonal rabbit anti-human IgG (Dako, Carpinteria, CA, USA, cat. no. A042301) conjugated with Cy3 dye (GE Healthcare, Chicago, IL, USA, cat. no. GEHPA23001) for two hours. After antibody incubation, several more rounds of washing in SAB and ultra-pure water were performed prior to drying of the microarrays by centrifugation. The microarrays were scanned using an Innoscan 710 microarray scanner (Innopsys, Carbonne, France) at 532 nm, with scan settings set to 100 PMT gain and 5 µm resolution.

The raw image files were processed in SpotXEL (version 3.4.2; SICASYS Software GmbH, Germersheim, Germany), where each spot was quantified and annotated to its corresponding protein. Local background intensities were subtracted from spot intensities to generate foreground signals. Further processing was done using foreground intensities, specifically the median, to minimize the impact of potential minor artifacts on a spot.

Further data processing was performed in Python (version 3.11; Python Software Foundation, Wilmington, DE, USA). Inter-slide normalization was performed using Cy3BSA control spots that were present on all arrays. Cy3BSA intensities across all slides were ranked and used to generate a reference intensity profile. An array-specific scaling factor for each microarray was calculated by comparing its Cy3BSA intensities to the reference intensity profile, and the factor was applied to all the intensity data of the respective microarray prior to downstream statistical analysis.

Following normalization, quadruplicate spot intensities were averaged, and the coefficient of variation (CV) across quadruplicates was calculated to assess reproducibility across replicate spots. Reactivities exceeding 20% CV were excluded. To identify elevated antibody reactivities, Z-scores were calculated for each protein on each array based on its reactivity intensity compared to the rest of that microarray. Only proteins with Z-scores greater than two, meaning their average intensity deviated more than two standard deviations from the specific microarray’s overall intensity distribution, were included for downstream analysis.

## 3. Results

### 3.1. Clinical Characteristics and Comparison of SLE Patient Subgroups

**Figure 1:**
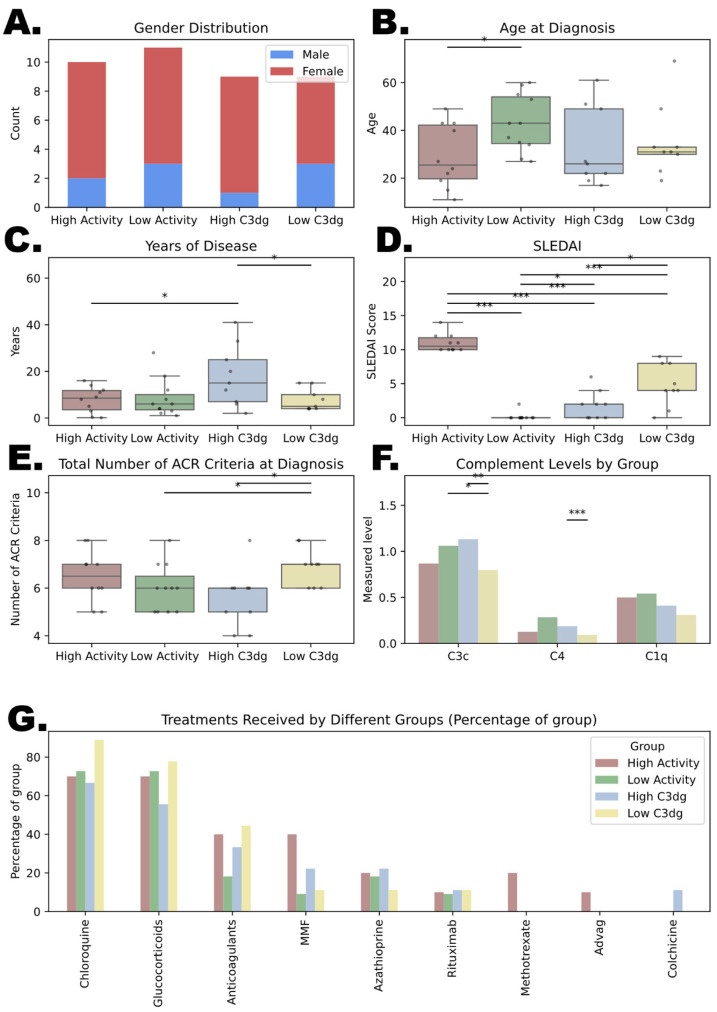
Clinical data across the four systemic lupus erythematosus (SLE) groups included in this study: high activity, low activity, high C3dg, and low C3dg. (**A**) Gender distribution within the groups; (**B**) age at diagnosis across groups; (**C**) disease duration between groups; (**D**) SLEDAI scores between the groups; (**E**) total number of ACR criteria fulfilled at diagnosis across groups (each specific criteria can be found in Supplementary File S1, Sheet 1); (**F**) complement levels, including C3c, C4, and C1q; (**G**) shows the treatment received by patients in different groups, expressed as the percentage of the specific group receiving each type of treatment. * *p* < 0.05, ** *p* < 0.01, *** *p* < 0.001.

The selected study cohort included 39 subjects allocated to four groups: high and low disease activity as measured by SLEDAI scoring, and high and low complement activity, measured by C3dg levels. Baseline and clinical and demographic characteristics of the cohort, as well as treatment information, can be found in Figure 1. Renal markers at inclusion showed eGFR values ranging from 38 to >90 mL/min. Six patients had eGFR below 60 mL/min, including two in the low-activity group, three in the high-C3dg group and one in the low-C3dg group. Proteinuria was present in 10 patients, with two to three affected patients per subgroup. Additional information including individual SLEDAI scores, ACR classification criteria, renal variables, complement measurements, and treatment information are provided in Supplementary File S1, Sheet 1.

### 3.2. Proteomic Profiling of Soluble Plasma Proteins

#### 3.2.1. Differential Protein Expression Analysis

Four replicates of each plasma sample from individual patients were analyzed by bottom-up proteomics. We identified and quantified 319 protein groups across all samples. After filtering away proteins identified in fewer than 50% of the samples (removing 42 proteins) and those identified by only a single unique peptide (removing 89 proteins), 188 protein groups quantified using a label-free approach remained for statistical data analysis. Protein group-level CV analysis showed good reproducibility across quadruplicate technical replicates, with an average intra-sample CV of 13.56% (±2.04).

**Figure 2:**
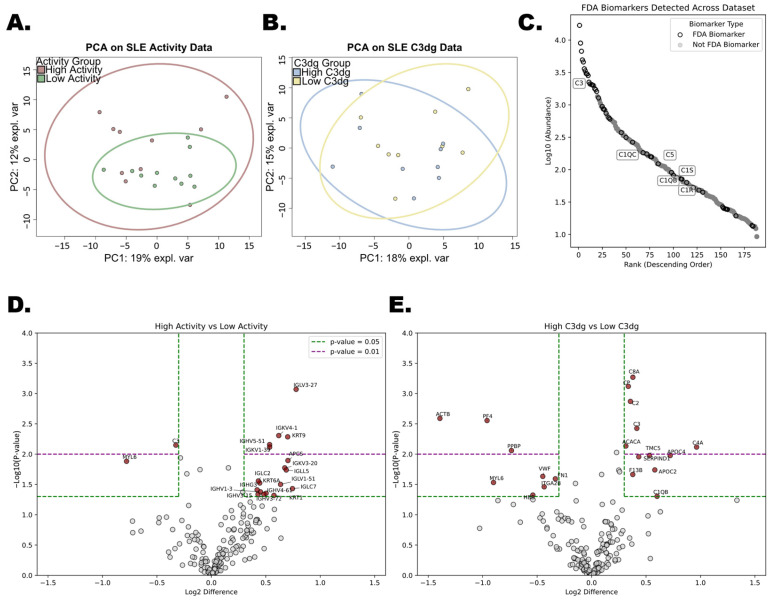
(**A**,**B**) Principal component analyses with 95% confidence ellipses illustrating sample distribution across component 1 and component 2 for both analyses; high versus low activity (determined by SLEDAI score) and high versus low C3dg. (**C**) Ranked abundance of the proteins detected across the systemic lupus erythematosus (SLE) cohort. Each protein identified in the SLE cohort is plotted in descending order of abundance on a logarithmic scale (Log10). Black circles represent identified proteins that are FDA-approved biomarkers, while gray dots represent the rest of the identified proteins. The labeled proteins are FDA biomarkers that are known to play important roles in the KEGG pathway for SLE. (**D**,**E**) Volcano plots showcasing the differential protein expression among SLE subgroups. (**D**) compares protein expression between SLE patients with high disease activity and low disease activity (determined by SLEDAI score). (**E**) Compares protein expression between SLE patients with high and low C3dg content in their blood. Significantly regulated proteins at two *p*-values (raw 0.05; 0.01) with Log2 fold-change over ±0.3 are highlighted in red.

To evaluate whether unsupervised clustering was sufficient to separate between patient groups, we performed principal components analyses (PCAs) for both comparisons; high versus low activity and high versus low C3dg. However, as shown in Figure 2A,B, no clear separation was observed across component 1 and 2, highlighting the need for a supervised sPLS-DA analysis to explore potential group-separating plasma proteomic patterns.

Next, to evaluate the clinical relevance of the identified proteins, we examined how many FDA-approved plasma and serum protein biomarkers were identified in our SLE cohort. As a reference, we utilized a list of 109 plasma and serum FDA biomarkers documented by Anderson et al. [18]. In our dataset, we successfully identified and quantified 39 of these biomarkers, as illustrated in Figure 2C; the biomarkers were acquired within less than three minutes of analysis time.

Finally, we performed differential expression analyses. In the comparison between SLE patients with high and low disease activity, 25 proteins were significantly differentially expressed (*p* < 0.05). Of these, 20 proteins were overabundant in the high-activity group, while five were more abundant in the low-activity group. For the high versus low C3dg comparison, 25 proteins showed significant differences in expression (*p* < 0.05). Among them, 16 were upregulated in the high-C3dg group, while nine were more abundant in the low-C3dg group. These results are visualized in Figure 2D,E. A full table of the identified proteins, their quantities and the statistical analysis can be found in Supplementary File S1, Sheet 5.

#### 3.2.2. Supervised Group Separation Analysis

To accommodate for the lack of evident patient group separation by PCA analysis, we performed a supervised sPLS-DA analysis to identify the proteins that contribute the most to group separation across the components selected by the model. For the comparison between the high- and low-SLEDAI groups, the model tuning deemed two components sufficient to capture the key variance. Component 1 was composed of 28 proteins and component 2 consisted of four proteins. The model for separation between high- and low-C3dg groups consists of a single component of 13 proteins. Model performance was assessed using repeated 10-fold (activity) and 9-fold (C3dg) cross-validation (75 repeats) and showed a balanced error rate (BER) of 0.234 for the activity model, indicating moderate classification performance, and 0.109 for the C3dg model, indicating good classification performance.

**Figure 3:**
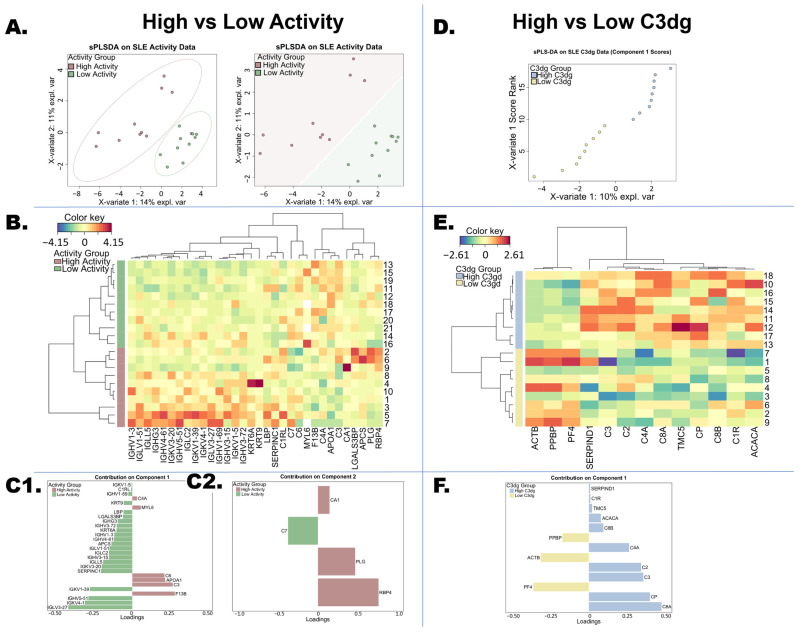
This plot shows two comprehensive sPLS-DA models highlighting the most discriminative features between the compared groups; on the left, SLE patients with high versus low disease activity, and on the right, SLE patients with high C3dg blood levels versus patients with low C3dg blood levels. (**A**) The left plot shows the separation of the high-activity patients compared to the low-activity patients based on the most important features as selected by the model. Around the datapoints for each group, confidence ellipses show where the true means of the populations from which the samples are drawn would be expected to lie with 95% confidence. The right plot shows the same datapoints as the left plot but with a prediction background, illustrating the decision boundaries and how new samples would be classified according to the model. (**B**) A clustered image map for SLE activity data. The colored matrix shows the relative expression of the proteins selected for the two components utilized in the sPLS-DA model. The rows of samples are sorted based on similarity in their expression profiles of the selected proteins. (**C1**,**C2**) illustrate the proteins (loadings) used for each component of the SLE activity model, along with the corresponding weight of each loading. These weights indicate the influence or contribution of each protein to the respective component. (**D**) shows the separation of patients grouped by their levels of C3dg, high and low. Since the model only uses one component to separate the samples, the component 1 value of each sample is plotted on the x-axis and the component 1 scores are ranked in ascending order on the y-axis. (**E**) shows a clustered image map based on SLE C3dg data. The colored matrix shows the relative expression of the proteins selected for the model’s components (in this case only one component), and the rows of samples are organized based on their similarity in expression of the selected proteins. (**F**) shows the loadings (proteins) selected by the model for component 1 and the weighted contribution of each loading to the component.

As shown in Figure 3A, the features selected by the sPLS-DA model show apparent separation between the high- and low-activity groups, with 95% confidence ellipses highlighting the group separation. To further illustrate the expression patterns of the separation-driving proteins, Figure 3B presents a clustered image map in which the samples are ordered by similarities in their expression of these proteins. The proteins selected for each component and their weight of contribution towards group separation between the groups are illustrated in Figure 3C1 for component 1 and Figure 3C2 for component 2.

For the C3dg model, the sPLS-DA model identified a single component as sufficient for capturing the main variance between the high and low groups. Figure 3D shows the apparent separation of the groups based on their expression of the key discriminatory proteins according to the model. Since the model resulted in a single component, the variate values are plotted on the x-axis and ranked in ascending order on the y-axis to highlight the separation. The expression of the key proteins of the model is displayed in a clustered image map on Figure 3E, while the proteins’ contributions to the separation are illustrated on Figure 3F.

#### 3.2.3. Functional Enrichment Analysis of the Four SLE Subgroups

**Figure 4:**
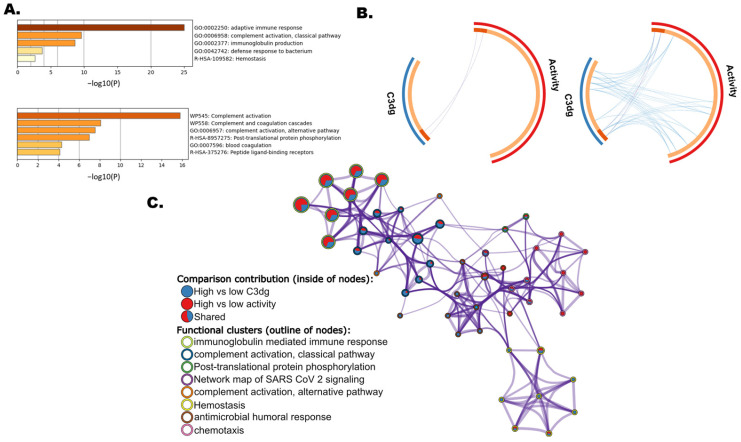
(**A**) Enriched terms and their significance among the most discriminatory proteins in SLE patients, comparing high versus low-SLEDAI groups (top) and high versus low-C3dg groups (bottom). (**B**) Overlapping Circos plots. Left: overlap in discriminatory proteins between the two comparisons, where purple lines connect identical proteins. Right: overlap based on functional similarity of proteins. Lines connect proteins that belong to the same enriched ontology terms. Only terms with fewer than 100 genes are included to avoid excessive linking. (**C**) Network of enriched biological terms across the two comparisons; high versus low SLE activity and high versus low C3dg. Each node represents an enriched term, and the size of a node is proportional to the number of discriminatory input proteins associated with the term. The inside of each node is a pie chart visualizing the proportion of proteins contributed by each comparison. Edges connect terms based on overlap in the proteins constituting the terms. Terms are grouped into functional clusters by hierarchical clustering, and the outline of each node is colored to visualize which cluster the enriched term belongs to. Each cluster is named after the most significantly enriched term within that cluster. A full overview of clusters and their enriched terms can be found in Supplementary File S1, Sheet 9. Enrichment analysis was performed using https://metascape.org [19].

For the discriminatory proteins selected by each sPLS-DA model, we performed a term enrichment analysis to identify biological pathways associated with the discriminating proteins. For the SLEDAI comparison, the proteins that were deemed most important for group separation by the sPLS-DA model’s first component were found to be significantly enriched in biological processes like adaptive immune response, classical complement activation, immunoglobulin production, defense against bacterium, and homeostasis; see Figure 4A.

For the comparison between patients with high and low levels of C3dg, the proteins responsible for most of the group separation according to the sPLS-DA model were found to be enriched in biological processes such as complement activation, complement and coagulation cascades, and alternative pathway complement activation; see Figure 4A.

### 3.3. Autoantibody and Inflammatory Profiling

**Figure 5:**
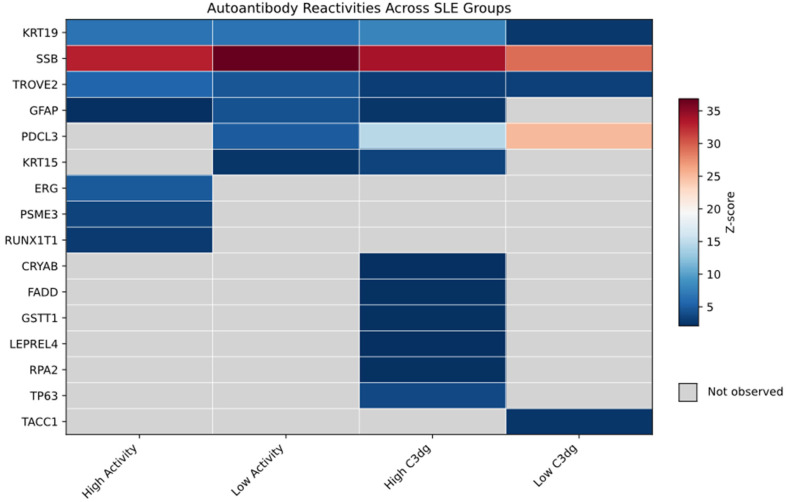
Autoantibody reactivities assessed by protein array profiling. Pooled samples were analyzed by protein microarrays. Signal intensities expressed as Z-scores are shown to enable comparison of autoantibody reactivity patterns across the four subgroups. Reactivities with intra-array CVs < 20%, and Z-scores ≥ 2 are displayed.

Exploratory autoantibody profiling was performed to provide an overview of autoantibody reactivities across the four cohort subgroups. Figure 5 summarizes all reactivities detected across the four groups as a heatmap of Z-score signals, highlighting shared and subgroup-specific autoantibody targets. Only proteins with intra-array CVs below 20% and Z-scores ≥ 2 are included in the heatmap. Across the four groups, 16 proteins were identified as autoantibody targets, of which three (KRT19, SSB, and TROVE2) were detected across all groups. Notably, the high-SLEDAI group has three unique reactivities compared to the other groups (ERG, PSME3, RUNX1T1), while the low-SLEDAI group did not exhibit any unique reactivities. A similar pattern was observed for the C3dg groups, where the high-complement-activation group showed six unique reactivities (CRYAB, FADD, GSTT1, LEPREL4, RPA2, TP63), while the low-C3dg group had just one unique reactivity (TACC1). Full RFU intensities, coefficients of variation (CV%), and Z-scores for all included autoantibodies are available in Table 1.

## 4. Discussion

The primary aim of this study was to characterize the plasma proteomics phenotype associated with C3dg-defined complement activity in SLE pathology while also assessing the feasibility of using rapid LC-MS/MS methods in a future clinical setting. By comparing proteomic profiles across two SLE patient groups defined by SLEDAI scores and C3dg levels, we aim to determine whether these markers reflect different or overlapping aspects of SLE pathophysiology. Our findings suggest that the two markers may capture related, but not identical, aspects of SLE.

Interestingly, the low-C3dg group had significantly higher SLEDAI scores than the high-C3dg group. This inverse relationship suggests that while complement activity, as measured by C3dg, is an established feature in the pathophysiology of SLE, high complement activation is not directly linked to higher disease activity as captured by the SLEDAI. This observation highlights the complexity of SLE and underscores the importance of understanding the potentially complementary insights into disease activity provided by the two markers. From a clinical perspective, the observation that high SLEDAI scores are not necessarily accompanied by high C3dg levels should not be interpreted as evidence that complement activation is clinically irrelevant. Rather, it suggests that C3dg may reflect a biological state that is not fully captured by current composite disease activity indices. To investigate the biological processes associated with SLEDAI scores and C3dg levels, we constructed two sPLS-DA models: one comparing SLE patients with high and low C3dg levels and another comparing patients with high and low disease activity based on SLEDAI. We used the most discriminative protein features from each sPLS-DA model to perform enrichment analyses to identify the biological pathways associated with each marker; see Figure 4A.

### 4.1. Proteomic Differences Associated with Disease Activity and Complement Activation

The high-SLEDAI group showed upregulation of 20 proteins and downregulation of 5 proteins compared to the low-SLEDAI group. Overall, these proteins reflect an increase in adaptive immune activity and systemic inflammation. The majority of the significantly upregulated proteins are immunoglobulin heavy and light chains (e.g., IGHV3-72, IGHV5-51, IGKV3-20, IGLV3-27, IGKV4-1, IGHV3-15) indicating increased B-cell activity and thus antibody production and immune complex-driven disease, which are known to be central aspects of SLE pathology [2,3]. Conversely, complement-related proteins (C3 and C6) were found to be significantly downregulated in the high-SLEDAI group, aligning with the established link between hypocomplementemia and SLE; however, as described earlier, low complement levels do not necessarily indicate breakdown through activation but may instead reflect low levels of synthesis [4].

The protein-level findings among significantly regulated proteins is supported by the functional enrichment analysis performed on the most discriminatory proteins identified by the sPLS-DA model. This analysis revealed enrichment of biological terms related to adaptive immune responses, immunoglobulin production, and complement activation via the classical pathway; see Figure 4A. Together, these findings indicate that activity captured by SLEDAI reflects classic adaptive immunity driven through antibody–antigen complexes and classical complement activation.

The protein signature in the plasma of patients with high C3dg levels primarily reflected complement involvement. Complement components such as C1R, C1QB, C2, C3, C4A, C6, C8A, and C8B were all significantly upregulated in the high-C3dg group compared to the low-C3dg group. Interestingly, this contrasts with the high versus low SLEDAI comparison, where complement components were downregulated in the high-activity group due to assumed consumption of complement components. However, in the C3dg group, we observe that patients with high complement activity, as indicated by high C3dg levels, may not exhibit hypocomplementemia. This duality highlights the potential of C3dg as a marker of actual complement activity as opposed to quantification of C3, C4 and CH50, which, as noted earlier, was incorporated into the SLICC criteria for SLE in 2012 [7,20].

In addition to complement components, we also observed an upregulation of coagulation-associated proteins. This is particularly relevant, as SLE increases the risk of developing thrombotic events by 25- to 50-fold compared to the general population [21]. Upregulated coagulation-associated proteins include F13B, PPBP, PF4, and SERPIND1. F13B (coagulation factor XIII subunit B) is part of a key protein involved in the stabilization of fibrin clots [22]. Similarly, PPBP (platelet basic protein) and PF4 (platelet factor 4), both CXC family chemokines known to be released after platelet activation in alpha granules, are elevated, suggesting enhanced clot formation [23]. The upregulation of these proteins within the high-complement-activity group may indicate a heightened thrombo-inflammatory environment in this patient group [24].

Interestingly, SERPIND1 (heparin cofactor II), a protein that acts as a cofactor for heparin and dermatan sulfate, and thus facilitates inhibition of thrombin, was also found to be upregulated [25]. This counteracts the pro-thrombotic actions of the previously described proteins, especially PF4, a protein that binds with high affinity to heparin and inhibits its anti-thrombotic functions [23]. This opposing regulation may indicate a compensatory relationship where both pro-thrombotic and anti-thrombotic mechanisms are upregulated in parallel. The increased levels of SERPIND1 could reflect a mechanism to combat the pro-thrombotic effects of the other proteins. To clarify the clinical implications on the thrombo-inflammatory changes observed in this patient population, further studies should be conducted to investigate the frequency of thrombotic events in SLE patients with high or low C3dg. Such a study could reveal whether these protein expression alterations skew towards a more pro- or anti-thrombotic environment in the patients. If validated, a high C3dg phenotype could help identify patients at higher risk of complement-mediated tissue injury or thrombo-inflammatory complications.

Again, the protein-level patterns align with the enriched term analysis performed on the features deemed the most discriminatory by the sPLS-DA model, which revealed enrichment of biological processes related to complement activation, specifically through the alternative pathway, as well as coagulation and blood coagulation cascades. Together, the protein-level findings and enriched terms both support the hypothesis that C3dg levels reflect active complement biology and may be associated with coagulation pathways, reflecting an altered thrombo-inflammatory environment within this patient group [26,27,28].

Notably, only a limited overlap was observed between the most discriminatory protein features identified in the SLEDAI- and C3dg-based sPLS-DA models. In contrast, a substantially higher overlap between enriched terms was observed; see Figure 4B. Importantly, SLEDAI and C3dg comparisons should not be interpreted as fully independent biological contrasts, as SLEDAI contains serological components, including complement levels. Therefore, some inherent overlap in biological processes reflected by the two markers is expected. This aligns with our observation of a partial overlap in immune-complement biology, while the C3dg-based comparison may also emphasize complement and coagulation-related pathways.

### 4.2. Clinical Relevance of Rapid Mass Spectrometry Analyses

In this study, we utilized a 500 SPD method (2.9 min per sample) to identify 319 proteins, with 188 remaining after filtration steps across 39 plasma samples. Each sample was analyzed in quadruplicate with a mean CV of 13.56% (±2.04). Furthermore, we identified 39 of 109 known FDA-approved plasma protein biomarkers.

Historically, clinical adoption of LC-MS/MS-based assays has been limited by numerous challenges. Thomas et al. presented several barriers hindering the implementation of LC-MS/MS in a clinical setting [29]. Among these challenges are the manual nature of workflows, lack of automation, and low sample throughput. These specific challenges are rapidly being addressed. A key example is the high-throughput mass spectrometry analysis deployed in this study. It demonstrates that increased sample throughput can be achieved while maintaining high replicate reproducibility and clinical relevance of identified proteins. Automation of sample preparation is also rapidly improving. Kverneland et al. describes a fully automated sample preparation workflow from protein digestion and sample loading on Evotips with minimal manual involvement [30]. This workflow allows up to 192 samples to be prepared in less than 7 h while improving the reproducibility of the sample preparation. With continued advances in automation and instrument throughput, implementation of routine clinical mass spectrometry is becoming increasingly realistic. However, despite these advances, broad LC-MS/MS-based profiling, such as that applied in this study, is currently better suited for research and translational laboratories than for routine clinical diagnostics. Broad untargeted profiling requires complex bioinformatic pipelines and careful interpretation of high-dimensional data and is therefore more suited for hypothesis generation and biomarker discovery requiring further validation. Therefore, a more realistic route toward clinical LC-MS/MS implementation is the discovery of smaller panels of validated protein signatures in research laboratories, which can be translated into targeted assays for rapid profiling and quantified in clinical settings to support diagnostics or decision-making. From a cost perspective, Thomas et al. highlight the initial expense of implementing a functional LC-MS/MS platform as a major barrier for clinical implementation due to the expensive instrumentation, potential renovation costs, service, maintenance, and the need for highly trained technical personnel [29]. However, once implemented, as instrument throughput and automation improve, the per-sample costs can be as low as a few dollars depending on the sample preparation strategy used [31].

### 4.3. Autoantibody Profiling Identifies a Subset of Antigens Related to the Disease Group

In addition to the quantitative LC-MS/MS proteome analysis, our exploratory autoantibody profiling provided a snapshot of the humoral immune response within our cohort. It is important to emphasize that the pooled nature of the setup (*n* = 5 per group) limits the ability to draw definitive quantitative conclusions across the groups. The pooled approach inevitably masks individual variance and may allow individual autoreactivity to disproportionately influence the group profile. Thus, the antibody reactivities observed serve as an exploratory, hypothesis-generating overview of the autoantibody landscape in SLE, and clinical relevance relies on further experiments [13,32]. These exploratory findings should be validated in future studies potentially employing targeted microarrays focused on the proteins of interest identified in the current study.

Across all four groups, three proteins (KRT19, SSB, and TROVE2) were consistently detected as autoantibody targets. Of these, the ANAs SSB and TROVE2 have been widely reported as autoantigens in SLE. Specifically, SSB is referenced in 94 PubMed abstracts in relation to SLE, and TROVE2 appears in 163, according to the human autoantigen text-mining database AAgAtlas [10]. The other antigen common to all groups (KRT19) has not previously been reported in AAgAtlas or other studies that we are aware of as an SLE autoantigen, and thus its role in SLE pathology is worth investigating further. KRT19 is an intracellular, cytoskeletal protein that is expressed primarily in epithelial cells. Hence, the cause of loss of tolerance against it is likely similar to that of the classical ANAs seen in SLE, where proteins are exposed to the immune system following tissue damage leading to subsequent loss of tolerance and, ultimately, inflammation and formation of immune complexes.

Beyond the autoantibodies that were common across all four groups, our profiling also revealed autoantibodies that were only present within specific subgroups of patients. While definitive classification of these reactivities being group-specific would be premature, they serve as interesting exploratory targets that may be validated as group-specific in future studies. Notably, the high-C3dg group had multiple antigens that were uniquely reactive in that group (CRYAB, FADD, GSTT1, LEPREL4, RPA2, and TP63). These are all intracellular proteins, and thus their role as reactive autoantigens is likely a reflection of unusual availability to the immune system, rather than a disease-driving mechanism. Similarly, three autoreactivities are unique to the high-SLEDAI group (ERG, PSME3, and RUNX1T1) and one to the low-C3dg group (TACC1), and these are also all intracellular proteins.

The broader range of autoantibody reactivities in the high-C3dg and high-SLEDAI groups may indicate a higher degree of immune-mediated tissue damage in these groups. However, as stated already, due to the pooled design of the microarray experiment, the analysis is exploratory and requires validation.

Autoantibodies against complement components or their regulators could explain the elevated C3dg levels in this patient subgroup. Specifically, anti-C3 antibodies have been observed in lupus nephritis and have been shown to interfere with the ability of factor H and CR1 to regulate C3b activity [33]. However, our study was designed to characterize the proteomic phenotype associated with high C3dg rather than the cause of C3dg elevation, and these specific anti-complement antibodies were not measured. Future studies should investigate the relationship between the presence of anti-complement antibodies and high C3dg levels.

The main limitation of this study is the small cohort size, particularly after division into mutually exclusive subgroups. This limits the statistical power and increases the risk of false-positive findings in both the differential protein expression analysis and the feature selection performed in the sPLS-DA models. For this reason, the proteins and enriched pathways identified here should be interpreted as hypothesis-generating candidate signatures rather than validated biomarkers. Independent validation is required in larger cohorts to determine the reproducibility of our findings. Another limitation to the interpretability of this study is that the cohort is predominantly composed of individuals of Caucasian descent. Future studies could therefore investigate whether the observed proteomic and autoantibody patterns are consistent across different ethnicities. Furthermore, several clinical variables may influence plasma profiles, including age, disease duration, treatment exposure, and renal involvement. Because of the small subgroup sizes, the present study did not adjust for these factors. However, clinical laboratory data are available in Figure 1 as well as Supplementary File S1, Sheet 1.

## 5. Conclusions

In this study, we applied high-throughput plasma proteomics and autoantibody profiling to investigate molecular differences between systemic lupus erythematosus (SLE) patients stratified by disease activity (SLEDAI) and complement activity (C3dg). Our findings suggest that disease activity, as captured by SLEDAI scores, and complement activation, as measured by C3dg, are associated with overlapping yet non-identical plasma proteomic patterns. Elevated SLEDAI scores were primarily associated with adaptive immune activation and increased immunoglobulin production, whereas high C3dg levels were linked to enhanced complement activity and engagement of coagulation pathways.

The study also demonstrates the technical feasibility and translational potential of rapid, high-throughput proteomic workflows. Using short LC-MS/MS gradients, we identified disease-relevant plasma proteins while maintaining strong technical reproducibility, supporting the future potential of the technology in clinical testing or diagnostic approaches. The integration of proteomic profiling into clinical workflows may ultimately contribute to precision medicine approaches that better capture disease heterogeneity and guide individualized treatment strategies in SLE.

Overall, C3dg may provide complementary information to conventional disease activity indexes and warrants further evaluation as a marker of complement-active SLE biology. The proteomic and autoantibody findings presented in this study serve as exploratory signatures that require validation in larger independent cohorts.

## Figures and Tables

**Table 1:** Antibody reactivities observed across four groups: high SLEDAI, low SLEDAI, high C3dg and low C3dg. The table summarizes RFU (relative fluorescent units) intensities, CV% and Z-scores of antibody reactivities observed across quadruplicate spots per sample. Antibody reactivities with a CV above 20% and a Z-score smaller than 2 were filtered out.

	High SLEDAI			Low SLEDAI			High C3dg			Low C3dg		
Protein	Intensity	CV%	Z-Score	Intensity	CV%	Z-Score	Intensity	CV%	Z-Score	Intensity	CV%	Z-Score
KRT19	3515.12	4.12	3.02	5156.02	2.54	6.58	8809.14	2.61	8.01	3006.21	1.13	2.63
SSB	33,351.87	6.66	32.96	27,316.84	15.06	36.83	35,460.84	10.02	33.84	28,751.76	10.69	29.12
TROVE2	6208.82	14.87	5.72	3654.46	9.17	4.53	3704.48	12.90	3.06	3635.34	10.69	3.27
GFAP	2678.36	4.56	2.18	3463.14	1.95	4.27	3205.89	7.15	2.58	--	--	--
PDCL3	--	--	--	3988.64	9.43	4.99	15,623.90	2.88	14.61	24,871.29	19.22	25.13
KRT15	--	--	--	2176.56	6.02	2.51	4003.11	2.65	3.35	--	--	--
ERG	5321.56	8.81	4.83	--	--	--	--	--	--	--	--	--
PSME3	3899.16	10.01	3.40	--	--	--	--	--	--	--	--	--
RUNX1T1	3268.60	8.74	2.77	--	--	--	--	--	--	--	--	--
CRYAB	--	--	--	--	--	--	2763.18	3.90	2.15	--	--	--
FADD	--	--	--	--	--	--	2851.62	4.64	2.23	--	--	--
GSTT1	--	--	--	--	--	--	2840.69	4.68	2.22	--	--	--
LEPREL4	--	--	--	--	--	--	2692.71	16.73	2.08	--	--	--
RPA2	--	--	--	--	--	--	2974.33	10.91	2.35	--	--	--
TP63	--	--	--	--	--	--	4390.30	7.74	3.72	--	--	--
TACC1	--	--	--	--	--	--	--	--	--	2952.73	6.52	2.57

## Data Availability

The mass spectrometry proteomics data have been deposited to the ProteomeXchange Consortium via the PRIDE partner repository with the dataset identifier PXD066214.

## Supplementary Materials

The following supporting information can be downloaded at: https://www.mdpi.com/article/10.3390/biomedicines14061408/s1, File S1: Mass spectrometry, File S2: Microarray.
